# *Poria cocos* polysaccharide prevents alcohol-induced hepatic injury and inflammation by repressing oxidative stress and gut leakiness

**DOI:** 10.3389/fnut.2022.963598

**Published:** 2022-08-17

**Authors:** Yue-hang Jiang, Lei Wang, Wei-dong Chen, Yu-ting Duan, Ming-jie Sun, Jia-jing Huang, Dai-yin Peng, Nian-jun Yu, Yan-yan Wang, Yue Zhang

**Affiliations:** ^1^School of Pharmacy, Anhui University of Chinese Medicine, Hefei, China; ^2^MOE-Anhui Joint Collaborative Innovation Center for Quality Improvement of Anhui Genuine Chinese Medicinal Materials, Hefei, China; ^3^Institute of Pharmaceutics, Anhui Academy of Chinese Medicine, Hefei, China; ^4^Institute of Conservation and Development of Traditional Chinese Medicine Resources, Hefei, China

**Keywords:** *Poria cocos*, ALD, NF-κB, CYP2E1, polysaccharide

## Abstract

Alcoholic liver disease (ALD) is a major worldwide chronic liver disease accompanied by hepatic inflammation, gut leakiness, and abnormal oxidative stress. Our previous study demonstrated substantial hepatoprotective activity of the active *Poria cocos* polysaccharide (PCP-1C). The present study explored whether PCP-1C protects against ALD among hepatic inflammation, gut leakiness, and abnormal oxidative stress. The results showed that PCP-1C significantly improved alcohol-induced liver injury by decreasing serum biochemical parameters, alleviating hepatic steatosis, and reducing lipid accumulation caused by ALD. Moreover, PCP-1C treatment reduced hepatic inflammation by inhibiting the toll-like receptor 4 (TLR4)/nuclear factor-kappa B (NF-κB) signaling pathway and also improved hepatocyte apoptosis by inhibiting the cytochrome P450 2E1 (CYP2E1)/reactive oxygen species (ROS)/mitogen-activated protein kinases (MAPKs) signaling pathway. Regarding intestinal protection, PCP-1C could repair the intestinal barrier and reduce lipopolysaccharide (LPS) leakage. Generally, PCP-1C exerts a positive therapeutic effect on ALD, which may play a pivotal of decreasing inflammatory factor release, inhibiting oxidative stress and apoptosis, and improving intestinal barrier injury.

## Introduction

Alcoholic liver disease (ALD) is a toxic liver injury caused by long-term heavy drinking, which can progress from alcoholic fatty liver to alcoholic steatohepatitis and even hepatocellular carcinoma (1, 2). In 2020, alcohol-related deaths accounted for 3% of the total global death toll (3), highlighting the health problems caused by alcohol consumption. Currently, the main treatments for ALD include alcohol abstention, nutritional support, and treatment with chemical drugs. However, Complications such as alcohol withdrawal (4), and adverse reactions such as jaundice are also evident (5).

Oxidative stress and inflammation are essential mechanisms in ALD development. During ALD, the alcohol oxidation system of liver microsomes is activated. Its main metabolic enzyme, cytochrome CYP2E1 (CYP2E1), accelerates the metabolism of alcohol and produces excess reactive oxygen species to stimulate the oxidative stress injury (6). Reactive oxygen species (ROS) produced by CYP2E1 activate the mitogen-activated protein kinases (MAPKs) pathway to induce hepatocyte apoptosis and accelerate the ALD process (7, 8). Moreover, the lipopolysaccharide (LPS)-induced inflammatory pathway is another critical pathogenesis of ALD. Long-term alcohol consumption increases intestinal permeability, promotes LPS, a toxic product of intestinal flora, to enter the blood and liver, and binds with the TLR4 receptor on the surface of the Kupffer cell membrane (9–11). TLR4 activation activates NF-κB inflammatory pathway to accelerate the release of inflammatory cytokines, such as tumor necrosis factor-α (TNF-α), interleukin-6 (IL-6), and interleukin-1-β (IL-1β), aggravating hepatic inflammation and even systemic injury (12). Therefore, preventing alcohol-induced hepatic injury and inflammation by inhibiting oxidative stress and gut leakiness is a potential therapeutic approach to treat ALD.

*Poria cocos* (Schw.) Wolf, a functional edible and medicinal fungus, has been widely used in East Asia for centuries for its diuretic, sedative, and tonic effects (13–15). The main active components of *Poria cocos* are the polysaccharides (16), which have biological activities such as anti-oxidation (17), immune regulation, anti-inflammation field (18), and liver protection (19, 20). We have reported that *Poria cocos* polysaccharide (PCP) may inhibit the expression of CYP2E1 and suppress NF-κB inflammatory pathway to protect against ALD (21). In our previous study, a homogeneous *Poria cocos* polysaccharide (PCP-1C) was purified based on the hepatoprotective activity, which was composed of galactose, glucose, mannose, and fucose in a molar percentage of 43.5: 24.4: 17.4: 14.6. PCP-1C improves liver tissue damage in carbon tetrachloride (CCl_4_)-treated mice and relieve oxidative stress and inflammation (22). However, it is unclear whether PCP-1C has a protective effect on ALD. Therefore, this study aims to investigate the protective effect of PCP-1C on ALD, exploring whether PCP-1C is protective by reducing the release of inflammatory factors, inhibiting oxidative stress and apoptosis, and improving gut leakiness.

## Materials and methods

### Preparation of *Poria cocos* polysaccharide

According to the reported methods (22), the polysaccharides were defatted with 95% alcohol, extracted by distilled water and alcohol precipitation method, and the precipitated polysaccharides were deproteinized by the Sevag method, followed by the dialysis (molecular weight cutoff: 3,500 Da) and freeze-dried to obtain crude polysaccharides, which was then purified by anion exchange, and the washed fractions were retained. After dialysis (molecular weight cutoff: 3,500 Da) and freeze-drying, the washed polysaccharide was obtained. Then the washed polysaccharide was separated by a gel column and detected by phenol sulfuric acid method, with the third component being retained.

### Experimental animals

Ten-weeks-old specific pathogen free (SPF)-grade male C57BL/6N mice (22–25 g) were purchased from the Laboratory Animal Center of Anhui Medical University (Hefei, China, SCXK-2020-001). All animal experimental procedures were approved by the Experimental Center of Anhui University of Chinese Medicine (AHUCM-mouse-2021034). Mice were routinely fed with a free diet and water, maintained at room temperature (24 ± 2)^°^C, relative humidity 55 ± 5%, 12 h dark-light cycle, and adaptive feeding for 1 week.

### Animal treatment

ALD of the mice was replicated according to the Gao-Binge model (23, 24). Sixty-eight C57BL/6N mice were randomly divided into control group, model group, PCP-1C high-dose group (100 mg⋅kg^–1^), middle-dose group (50 mg⋅kg^–1^), low-dose group (25 mg⋅kg^–1^), and Bifendate group (DDB 200 mg⋅kg^–1^). The control group was given a control liquid diet all the time. In contrast, the other groups were given a 0, 1, 2, 3, and 4% alcoholic liquid diet for the first 5 days as the adaptive feeding, followed by a 5% Lieber-DeCarli alcoholic liquid diet for 10 days (Daitz Biotechnology, Co., Ltd.). PCP-1C and DDB (Wanbond Pharmaceutical Group Ltd.) was administered to the mice by gavage from day 6 until the end of the study period. On day 16, experimental mice were gavaged with a single dose of 31.5% ethanol (5 g⋅kg^–1^ body weight) in the early morning and sacrificed 9 h later. Pair-fed control mice received maltodextrin (9 g⋅kg^–1^ body weight) (Supplementary Figure 1). All mice were sacrificed by cervical dislocation, and the blood, liver, and ileum were collected. Serum samples were collected after centrifugation at 3,500 rpm at 4°C for 15 min. Partial liver and ileum tissues were fixed in 10% formalin solution for histological analysis, and the remaining tissues were immediately frozen at –80^°^C for further study.

### Biochemical analysis

The serum levels of alanine transaminase (ALT), aspartate transaminase (AST), alkaline phosphatase (AKP), total cholesterol (TC), and triglyceride (TG) were measured according to the kit (Nanjing Jiancheng Bioengineering Institute, China). The hepatic levels of malondialdehyde (MDA), superoxide dismutase (SOD), glutathione peroxidase (GSH-PX), and myeloperoxidase were detected according to the kit (Nanjing Jiancheng Bioengineering Institute, China). The serum levels of LPS, diamine oxidase (DAO), d-lactate (D-LA), IL-1β, IL-6, TNF-α, and nuclear factor E2-related factor 2 (Nrf2) were determined by ELISA (Quanzhou Ruixin Biological Technology Co., Ltd., China).

### Histopathological analysis

The liver tissues and ileum tissues were processed for hematoxylin and eosin (H&E) staining. The fixed tissues were washed 12 h with water, dehydrated and embedded, and made into paraffin tissue blocks. Then tissue sections were made (4 μm), dewaxed, hydrated, stained, and finally dehydrated, transparent, sealed, and photographed. Frozen liver tissues cut to 8 μm in thickness were preserved in 3.7% formaldehyde for 10 min, followed by Oil Red O staining.

### Immunofluorescence

The fixed sections were removed and rinsed after dewaxing. Goat serum was added and sealed in a wet box at 25^°^C for 15 min. Diluted F4/80 (Abcam, Cambridge, England), p-NF-κB P65 (Cell Signaling Technology Inc., United States), and occludin (Baaode Biological Technology Co., Ltd., Nanjing) primary antibodies were added and placed in a wet box at 4^°^C for 12 h, then removed and soaked in PBS (5 min × 3 times). Fluorescence secondary antibody (room temperature, 60 min) was added and washed in PBS for 5 min, thrice. 4’, 6-diamidino-2-phenylindole (DAPI) was added and immersed in PBS for 5 min × 3 times. Finally, an anti-fluorescence quenching agent was added, and the tablet was sealed. The staining was observed under a fluorescence microscope and photographed (Leica, Germany).

### Western blotting

Fresh liver tissue (100 mg) was taken and ground using liquid nitrogen. The total protein content was extracted from the liver tissues using a total protein extraction kit containing protease inhibitors (Biyuntian Biotechnology Co., Ltd., China) and phosphatase inhibitors (Biyuntian Biotechnology Co., Ltd., China) according to the manufacturer’s instructions. The protein concentration was determined by the BCA method (Biyuntian Biotechnology Co., Ltd., China). Total protein was separated by 10% sodium dodecyl sulfate polyacrylamide gel electropheresis and transferred to polyvinylidene fluoride membranes. After blocking with 5% skim milk in Tris-buffered saline (1x) for 2 h, the membranes were probed with appropriate primary antibodies at 4°C overnight and then detected by HRP-labeled anti-rabbit or HRP-labeled anti-mouse secondary antibodies (Zhongshan Jinqiao Biotechnology Co, Ltd., China). The antigen-antibody complex was detected by an enhanced ECL reagent (Biyuntian Biotechnology Co., Ltd., China). After exposure, Image J was used for grayscale analysis.

### Immunohistochemical

The liver tissue was sectioned in paraffin with a section thickness of 5 μm, followed by dewaxing, hydration, and antigen repair. The serum was used to seal the slices at 37^°^C for 30 min, and the slices were incubated with primary antibody overnight at 4^°^C Then, a DAB working solution was added, and the images were observed under the microscope.

### Intestinal permeability analysis

Intestinal permeability was detected by the FITC-Dextran tracer. After the mice were sacrificed, 8 cm of ileum sample was taken from the mice. Then, one end of the ileum was tied with the thread while 100 μL, 20 mg/mL FITC-Dextran (Aladdin Industrial Co., United States, 4,000 kDa) was injected into the other end. The ileum was tied, followed by incubation in 2 mL Krebs-Henseleit bicarbonate (KHBB) buffer at 37°C for 20 min. FITC-Dextran concentration in KHBB buffer solution was analyzed at 490 nm emission wavelength and 530 nm absorption wavelength (Hitachi Limited, Japan).

### Statistics analysis

Data are expressed as mean ± standard deviation (SD) of at least three replicates per assay. Analysis of variance (ANOVA) was carried out using the LSD test. Statistical significance was set to *P* < 0.05.

## Results

### *Poria cocos* polysaccharide decreased the alcohol-induced hepatic injury

**FIGURE 1:**
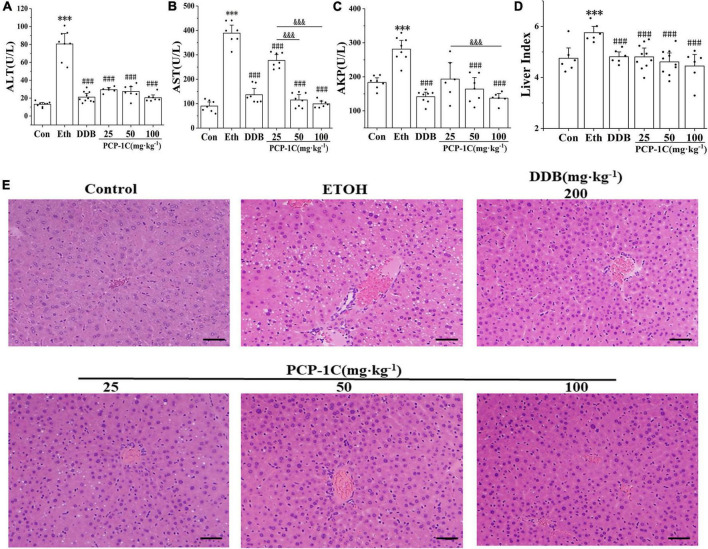
Effect of PCP-1C on general parameters of the ALD mice. **(A)** The serum levels of ALT, **(B)** AST, **(C)** AKP, and **(D)** mouse liver index. **(E)** Liver H&E staining of mice in each group (×200, scale 50 μm). The results are presented as mean ± SD (*n* ≥ 6). ^***^P < 0.001 vs. the control group; ^###^*P* < 0.001 vs. the model group; ^&⁣&&^*P* < 0.001 represents significance between PCP-1C groups.

C57BL/6N mice were orally administrated with different doses of PCP-1C to determine its protective effects against ALD. Compared to the control group, the serum levels of ALT, AST, AKP, and liver index were significantly increased in the model group. In contrast, DDB and PCP-1C at different doses significantly reduced the levels of ALT, AST, AKP, and liver index (Figures 1A–D). The H&E staining results indicated clear, structured liver lobules of the control group. The central veins ran neatly through the lobules and radiated from clear and irregular hepatic cords. However, microvesicular steatosis, inflammatory infiltration, necrosis, and hepatic lobule destruction were observed in the model group. In contrast, DDB and PCP-1C significantly improved liver injury, characterized by decreased hepatic steatosis and a significant alignment of hepatic cord radiation (Figure 1E). Taken together, these data suggest that PCP-1C can protect damaged livers, significantly reducing liver damage in ALD mice. In addition, the therapeutic effect of different doses of PCP-1C did not differ in terms of ALT and liver index.

### *Poria cocos* polysaccharide reduced alcohol-induced lipid accumulation in alcoholic liver disease mice

**FIGURE 2:**
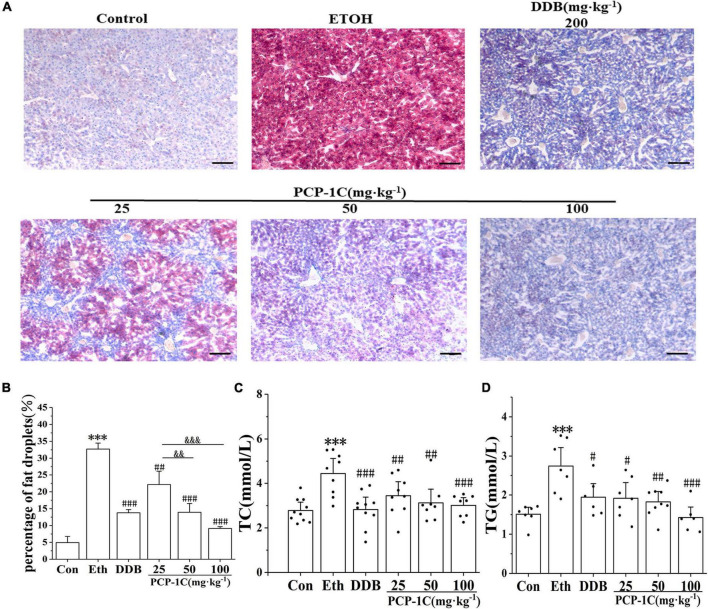
Effects of PCP-1C on hepatic steatosis and lipid accumulation in ALD mice. **(A)** Liver Oil Red O-staining (×200, scale 50 μm), **(B)** quantification of fat droplets from Oil Red O-staining, **(C)** serum TC levels, **(D)** serum TG levels. The results are presented as mean ± SD (*n* ≥ 6). ^***^P < 0.001 vs. the control group; ^#^*P* < 0.05, ^##^*P* < 0.01, ^###^*P* < 0.001 vs. the model group. ^&⁣&^*P* < 0.01, ^&⁣&⁣&^*P* < 0.001 represents significance between PCP-1C groups.

Oil Red O-staining is a standard indicator of liver lipid accumulation. Alcohol feeding increased liver lipid accumulation while PCP-1C decreased the lipid accumulation in alcoholic mice (Figures 1A,B). The levels of hepatic TC and TG further confirmed lipid accumulation in the liver tissue of the model group (Figures 2A,B). At the same time, PCP-1C significantly inhibited TC and TG levels, indicating that PCP-1C can reduce lipid accumulation in ALD mice (Figures 2C,D). However, there was no significant difference in the inhibitory effect of PCP-1C at each dose on TC and TG.

### *Poria cocos* polysaccharide reduced alcohol-induced hepatic inflammation in alcoholic liver disease mice

**FIGURE 3:**
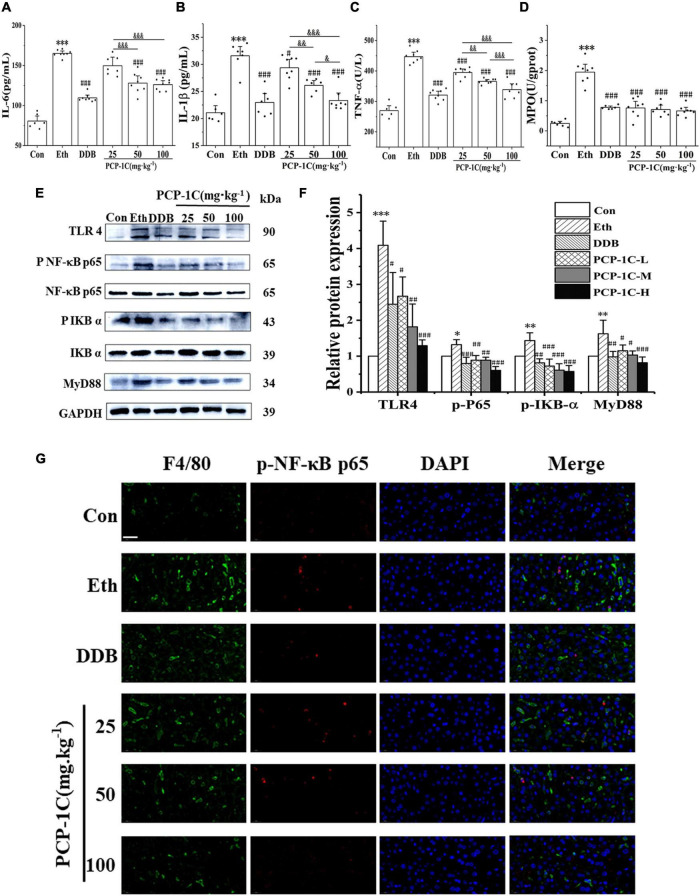
Effects of PCP-1C on inflammatory cytokines and related protein expression in ALD mice. **(A)** The serum levels of IL-6, **(B)** IL-1ß, **(C)** TNF-α, and **(D)** hepatic MPO levels. **(E)** Representative immunoblot images of TLR4/NF-κB related protein in liver tissue. **(F)** Relative protein levels and quantification of TLR4/NF-κB related proteins. **(G)** Immunofluorescence staining of liver tissue. (Green: F4/80, red: p-NF-κB p65, DAPI: The nucleus, ×400, scale 100 μm). The results are presented as mean ± SD. *P < 0.05, ^**^P < 0.01, ^***^P < 0.001 vs. the control group; ^#^*P* < 0.05, ^##^*P* < 0.05, ^###^*P* < 0.001 vs. the model group. ^&^*P* < 0.05, ^&⁣&^*P* < 0.01, ^&⁣&⁣&^*P* < 0.001 represents significance between PCP-1C groups.

After leakage of LPS into the blood, it binds to TLR4 receptors on Kupffer cells, stimulating myeloid differentiation factor 88 (MyD88) to phosphorylate IkappaB-alpha (IKB-α) and NF-κB, accelerating the release of inflammatory factors. DDB and PCP-1C significantly inhibited the abnormal elevation of TNF-α, IL-1β, IL-6, and inflammatory cell marker MPO induced by ALD (Figures 3A–D). The western blot results showed that alcohol feeding significantly increased the expression of TLR4/NF-κB signaling pathway-related proteins, including TLR4, MyD88, p-IKB-α/IKB-α, and p-NF-κB p65/NF-κB p65. However, DDB and PCP-1C significantly reversed this trend (Figures 3E,F). Further immunofluorescence staining showed the nuclear translocation of p-p65, an observation consistent with the western blotting results (Figure 3G). PCP-1C treatment could inhibit the nuclear translocation of p-NF-κB p65, suggesting a probable role of PCP-1C reducing inflammation by regulating TLR4/NF-κB signaling pathway. More importantly, PCP-1C significantly reduced the expression of F4/80 induced by ALD. These findings collectively prove that PCP-1C reduces inflammation by regulating TLR4/NF-κB signaling pathway. It should be noted that the effect of PCP-1C on IL-1ß and TNF-α is dose-dependent.

### *Poria cocos* polysaccharide decreased the oxidative stress in alcoholic liver disease mice

**FIGURE 4:**
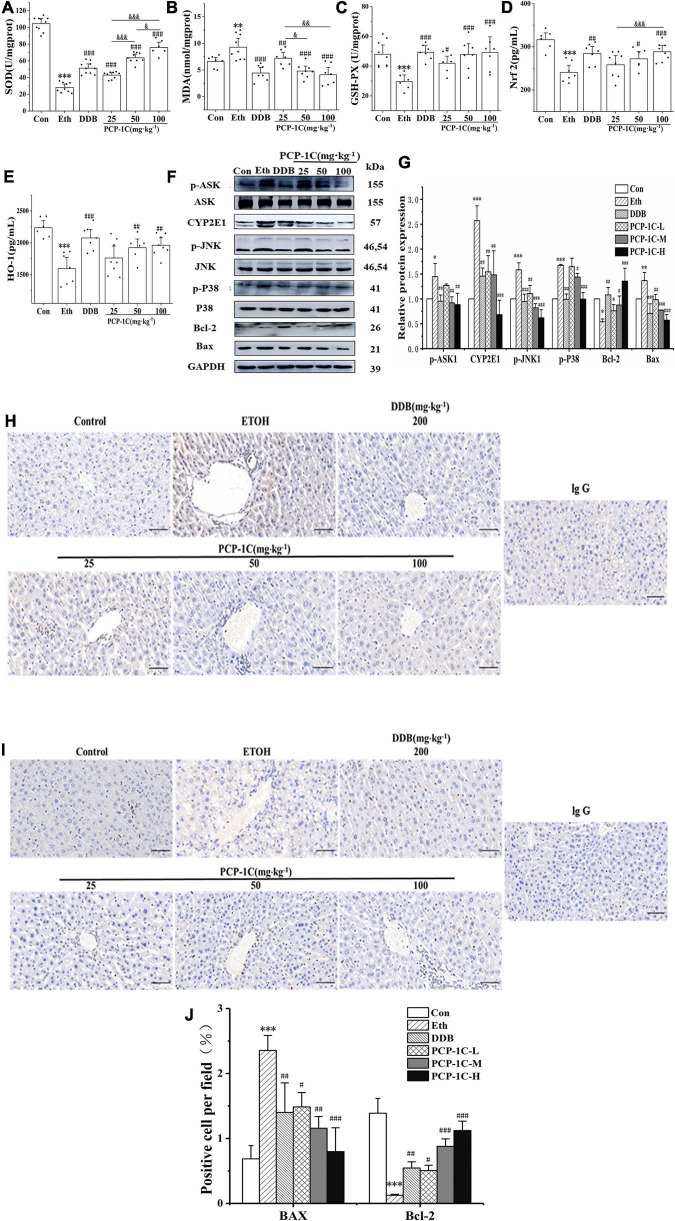
Effect of PCP-1C on oxidative stress and apoptosis in ALD mice. **(A)** Hepatic SOD levels. The serum levels of **(B)** MDA, **(C)** GSH-Px, **(D)** Nrf2, and **(E)** HO-1. **(F)** Representative immunoblot images of CYP2E1 and related proteins are involved in the apoptosis pathway in liver tissues. **(G)** Relative protein levels of CYP2E1 and related proteins involved in the apoptosis pathway. **(H–J)** Immunohistochemical staining of Bax and Bcl-2 in liver tissue. The results are presented as mean ± SD. *P < 0.05, ^**^P < 0.01, ^***^P < 0.001 vs. control group; ^#^*P* < 0.05, ^##^*P* < 0.05, ^###^*P* < 0.001 vs. model group. ^&^*P* < 0.05, ^&⁣&^*P* < 0.01, ^&⁣&⁣&^*P* < 0.001 represents significance between PCP-1C groups.

ROS accumulation increases the phosphorylation status of apoptosis signal-regulated kinase 1 (ASK1), which then promotes the phosphorylation of Jun N-terminal kinase-1 (JNK1) and P38 in the apoptotic pathway, altering the expression of apoptosis-related proteins and leading to abnormal apoptosis of hepatocytes. Alcohol feeding significantly reduced hepatic levels of antioxidant enzymes SOD and serum levels of GSH-Px, HO-1, and Nrf2, while increasing serum levels of the lipid metabolite MDA. PCP-1C significantly reversed the trends in the above indicators, indicating that PCP-1C has a protective effect against abnormal oxidative stress. Moreover, we observed that the impact of PCP-1C on SOD is dose-dependent (Figures 4A–E).

To gain insight into the mechanism of action of PCP-1C, western blotting was performed to study the relevant protein expression levels. The alcohol-feeding induced the hepatic CYP2E1 and activated the apoptotic pathway, indicated by increased protein levels of p-ASK, p-JNK, p-P38, and BCL2-Associated X (Bax), while the alcohol-feeding inhibited the expression of the apoptotic factor B-cell lymphoma 2 (Bcl-2). DDB and PCP-1C treatment significantly reversed the abnormal trend of the above proteins (Figures 4F,G). The immunohistochemical staining of Bax and Bcl-2 further confirmed the western blotting results. Collectively, these findings prove that PCP-1C inhibits oxidative stress and thus improves apoptosis induced by the downstream MAPKs pathway (Figures 4H–J).

### *Poria cocos* polysaccharide improved intestinal gut leakiness in alcoholic liver disease mice

**FIGURE 5:**
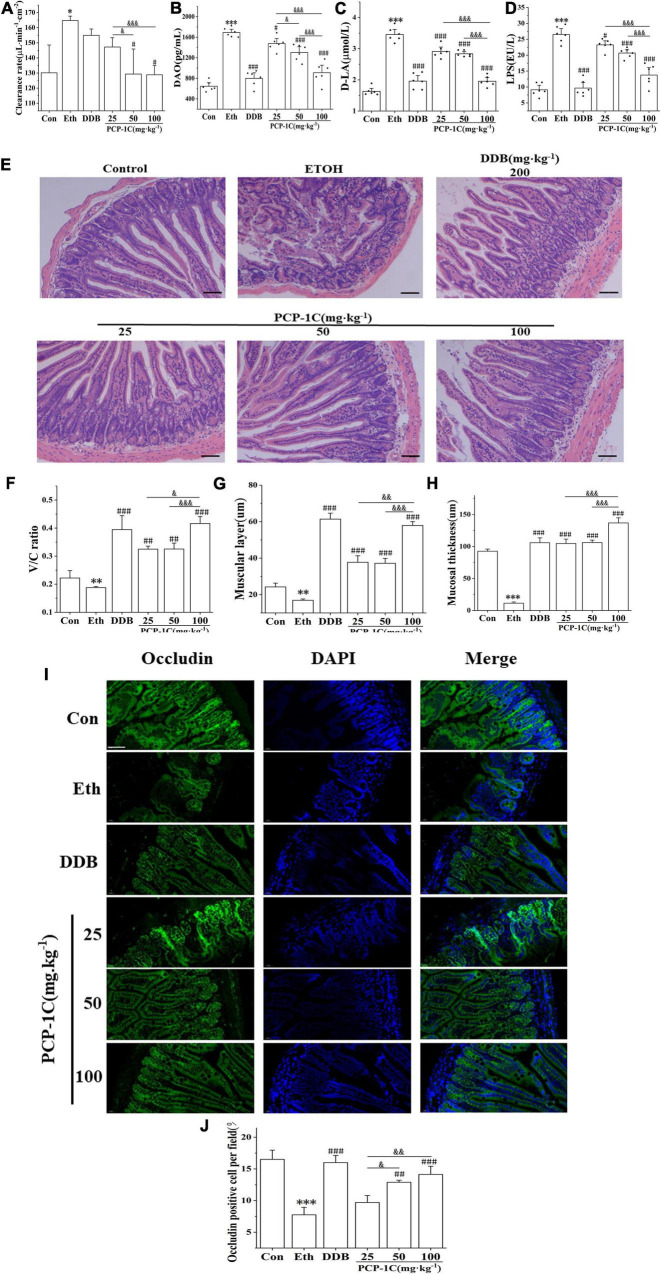
Effect of PCP-1C on the intestinal barrier injury in ALD mice. **(A)** The clearance rate of ileum per unit area in mice. **(B)** Serum levels of DAO, **(C)** D-La and **(D)** LPS, **(E–H)** H&E staining of the ileum. **(I,J)** Immunofluorescence staining of occludin in the ileum. (Green: occludin, blue: nucleus, ×400, scale 100 μ m). The results are presented as mean ± SD (*n* ≥ 6). *P < 0.05, ^**^P < 0.01, ^***^P < 0.001, vs. the control group; ^#^*P* < 0.05, ^##^*P* < 0.01, ^###^*P* < 0.001 vs. the model group. ^&^*P* < 0.05, ^&⁣&^*P* < 0.01, ^&⁣&⁣&^*P* < 0.001 represents significance between PCP-1C groups.

The absorption of LPS from the cell wall of intestinal Gram-negative bacteria into the blood and liver is the most classical pathogenesis of ALD, and gut leakiness is the crucial factor during this process. Compared to the control group, the alcohol-fed group had significantly increased ileal clearance (Supplementary Figure 2), abnormally elevated serum levels of DAO and D-LA, and unusually elevated serum levels of LPS, a product of intestinal flora. However, the above indicators were significantly reduced by PCP-1C and DDB treatment (Figures 5A–D). H&E staining of the ileum showed that the normal group had a complete intestinal mucosa structure with orderly and dense villi. In contrast, the alcohol-fed group had incomplete intestinal villi with partial intestinal mucosal epithelium exfoliation accompanied by inflammatory cell infiltration. PCP-1C and DDB could improve the intestinal mucosa integrity, alleviate intestinal injury and repair the disrupted barrier (Figures 5E–H). Immunofluorescence staining demonstrated that PCP-1C significantly increased the expression of occludin. These findings confirmed that PCP-1C could improve the intestinal mucosal barrier, reduce intestinal permeability, and inhibit hepatic inflammation (Figures 5I,J).

## Discussion

Improvement of living standards and social pressure has led to a yearly increase in the incidence of ALD. As the main metabolic site of the body, the liver plays a vital role in alcohol metabolism. LPS-induced inflammatory activation of Kupffer cells is core to the ALD pathogenesis (25). Long-term alcohol consumption causes an imbalance of intestinal flora and disrupts the integrity of the intestinal tract, accelerates the absorption of LPS into the blood, and binds to the TLR4 receptor on the surface of hepatic Kupffer cells (26). TLR4/MyD88 promotes the nuclear translocation of NF-kB, leading to increased release of the downstream inflammatory factors TNF-α, IL-1β, and IL-6, which accelerates ALD. Our study showed that PCP-1C treatment significantly inhibited the abnormal elevation of inflammatory factors, indicating that PCP-1C plays a protective role by inhibiting the TLR4/MyD88/NF-kB inflammatory pathway.

Overexpression-mediated oxidative stress damage in ALD is another important pathogenesis (27). SOD catalyzes the conversion of superoxide anion radicals to molecular oxygen and hydrogen peroxide (28). MDA is a lipid peroxidation product (29). GSH-PX uses glutathione as a reducing agent to break down lipid peroxides in the body to prevent cell membranes and other biological tissues from peroxidative damage (30), and Nrf2 is a critical factor in the cellular oxidative stress response that regulates the expression of related antioxidant pathways and the antioxidant enzyme HO-1 (31). We examined the changes in the above indexes. We found that PCP-1C significantly inhibited the content of MDA and improved the abnormal decrease of SOD, GSH-PX, Nrf2, and HO-1, suggesting a possible role of PCP-1C in improving the antioxidant capacity of the body (Supplementary Figure 3).

CYP2E1 is the most critical enzyme in alcohol metabolism and is the main producer of oxidation products in ALD (32). In the present study, we found that PCP-1C inhibited the overexpression of CYP2E1. The Wnt-β-catenin, TGF-β-Smads, and MAPK pathways are known to be regulated by CYP2E1 (33), and the MAPK pathway is more mature. Besides, the exogenous ROS generated by CYP2E1 overexpression activates ASK1. Phosphorylated ASK1 activates the downstream JNK1 and P38 signaling pathways. Thus, phosphorylated JNK1 and P38 promote the pro-apoptotic factor, Bax, in mitochondria, and inhibit the release of BCL-2, driving abnormal apoptosis in hepatocytes and aggravating the liver injury (7, 8, 34). In this case, PCP-1C may act as a hepatoprotective agent by regulating the expression of CYP2E1 enzymes and inhibiting the MAPKs apoptotic pathway. Similar to the studies that demonstrated that CYP2E1 is regulated by nuclear receptors (22, 35), we also found changes in the expression of ERRγ and RORα, which are responsible for regulating CYP2E1 (Supplementary Figure 4).

The effect of intestinal injury on the liver *via* the gut-liver axis is a critical pathogenic factor in ALD (36). Thus, this study examined the ameliorative influences of PCP-1C on intestinal damage. As we mentioned, leaky intestinal-derived LPS acts as a “second strike” to exacerbate hepatic steatosis and activate Kupffer to induce inflammation (9). DAO is a highly active intracellular enzyme in the upper villi of the human and mammalian small intestinal mucosa that reflects the integrity and degree of damage of the intestinal mechanical barrier (37). Simultaneously, D-LA is a product of intrinsic bacteria in the gastrointestinal tract that reflect changes in intestinal mucosal permeability (38, 39). Occludin is a tight junction protein; it will increase the permeability between intestinal epithelial cells and allow bacteria and endotoxin (especially LPS) to enter the body when it is damaged (40). PCP-1C significantly reduced LPS, DAO, and D-LA plasma levels, while occludin immunofluorescence staining demonstrated that PCP-1C improved intestinal barrier integrity. In brief, PCP-1C may alleviate alcoholic liver injury by intestinal barrier integrity, which is consistent with the studies by Sun et al. (41).

In summary, PCP-1C reduces LPS leakage by restoring intestinal damage, further inhibiting the hepatic TLR4/NF-κB pathway and inhibiting the CYP2E1/ROS/MAPKs signaling pathway, thereby protecting ALD. The intestinal flora digests polysaccharides in the small intestine, and the intestinal tract absorbs the degraded polysaccharides to take effect. Therefore, the intestinal flora is closely related to the activity of polysaccharides (42). This study confirms the hepatoprotective effect of PCP-1C on ALD, and the results suggest that PCP-1C may affect the balance of intestinal flora to reduce liver injury, which points to the direction of its following research. Hence, this study provides a scientific basis for treating ALD with PCP-1C and offers novel insight into the potential therapeutic strategy of PCP-1C-ALD.

## Data availability statement

The original contributions presented in this study are included in the article/Supplementary material, further inquiries can be directed to the corresponding authors.

## Ethics statement

The animal study was reviewed and approved by the Institutional Animal Care and Use Committee of the Laboratory Animals Center at Anhui University of Chinese Medicine, Hefei, China (No. AHUCM-mouse-2021034).

## Author contributions

Y-HJ and LW: research design, experiment performance, and manuscript writing. Y-TD and M-JS: experiment operation assistance. J-JH: literature search. N-JY, W-DC, and D-YP: co-supervision and provide funds. Y-YW and YZ: main supervision and research leadership. All authors contributed to the article and approved the submitted version.
