# Supplementary material for: Poria cocos polysaccharide prevents alcohol-induced hepatic injury and inflammation by repressing oxidative stress and gut leakiness
Source: Front Nutr. 2022 Aug 17;9:963598. doi: 10.3389/fnut.2022.963598 (PMC9428680; doi:10.3389/fnut.2022.963598)
Supplement: Supplementary file 1 [file Data_Sheet_1.PDF]

Supplementary Material

1 Supplementary Figures and Tables

1.1 Supplementary Figures

Supplementary data

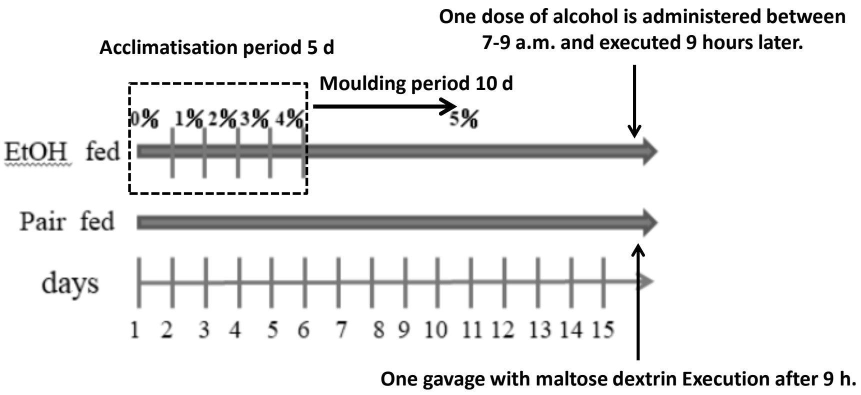

Supplementary FIGURE S1 **ALD modeling build details.**

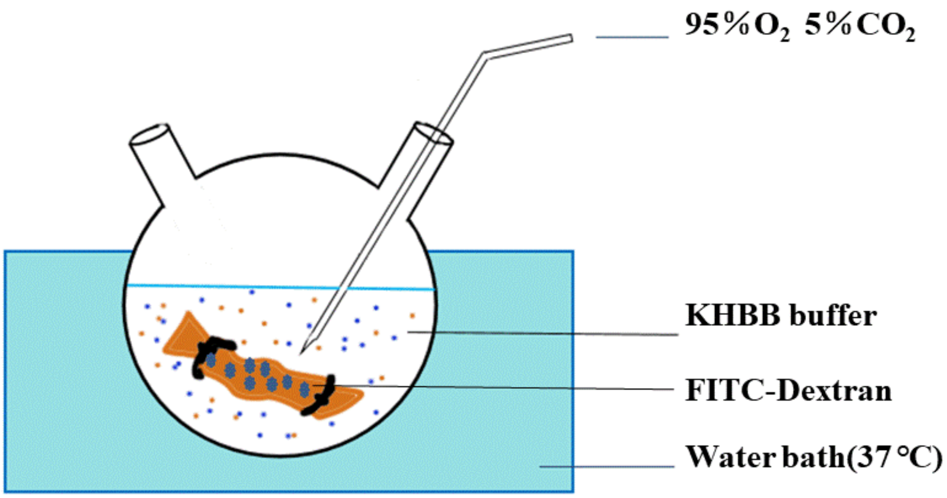

Supplementary FIGURE S2 FITC-Dextran tracer device diagram (System away from light)

The calculation formula of polysaccharide clearance rate is a formula.

$$C = \frac{[FD4]ser \times 1mL}{[FD4]muc \times A \times 30min}$$

C represents the clearance rate per unit area ($\mu L \cdot min^{-1} \cdot cm^{-2}$); A represents the surface area of the intestinal sac; A=лLD(cm^2), L represents the length of the intestinal sac (cm), D represents the diameter(cm); [FD4]ser represents the concentration of FD4 in the intestinal sac at 30 min; [FD4] muc represents the initial extracapsular solution concentration.

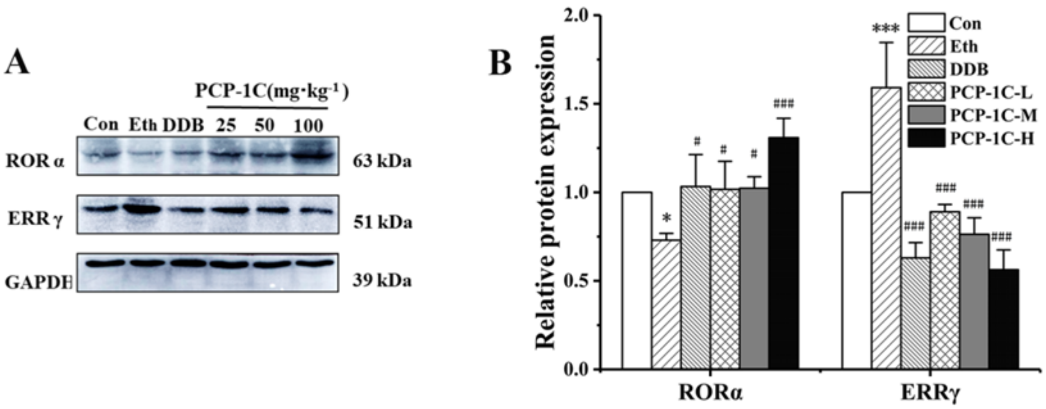

Supplementary FIGURE S3 **Effect of PCP-1C on nuclear receptors upstream of CYP2E1.**

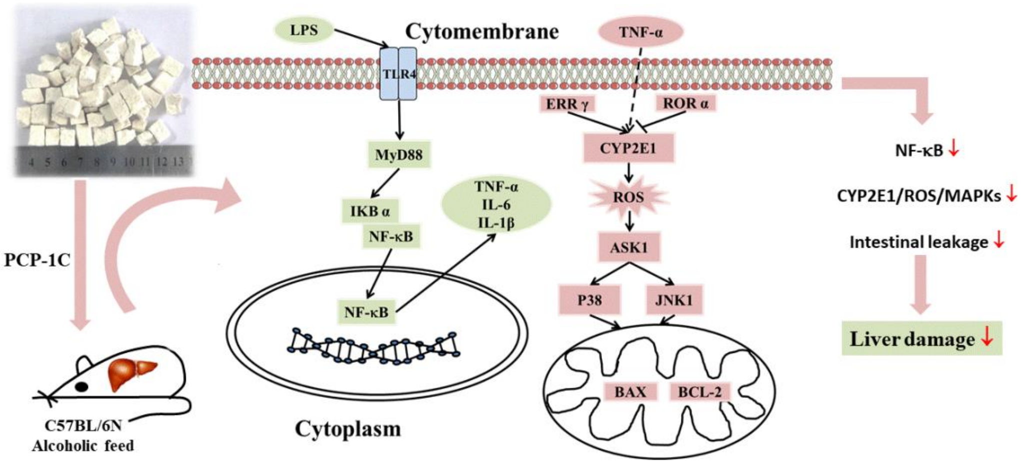

Supplementary FIGURE S4 **Schematic diagram of the effect of PCP-1C on ALD.**
